# Effect of S‐1 on survival outcomes in 838 patients with advanced pancreatic cancer: A 7‐year multicenter observational cohort study in Taiwan

**DOI:** 10.1002/cam4.2094

**Published:** 2019-04-18

**Authors:** Hsiang‐Lan Lai, Yen‐Yang Chen, Chang‐Hsien Lu, Chia‐Yen Hung, Yung‐Chia Kuo, Jen‐Shi Chen, Hung‐Chih Hsu, Ping‐Tsung Chen, Pei‐Hung Chang, Yu‐Shin Hung, Wen‐Chi Chou

**Affiliations:** ^1^ Division of Oncology Chang Gung Memorial Hospital at Kaohsiung Kaohsiung Taiwan; ^2^ Division of Oncology Chang Gung Memorial Hospital at Chiayi Chiayi Taiwan; ^3^ Division of Oncology Chang Gung Memorial Hospital at Linkou Taoyuan Taiwan; ^4^ College of Medicine Chang Gung University Taoyuan Taiwan; ^5^ Department of Hema‐oncology, Division of Internal Medicine Mackay Memorial Hospital Taipei Taiwan; ^6^ Division of Oncology Chang Gung Memorial Hospital at Keelung Keelung Taiwan; ^7^ Division of Hematology Chang Gung Memorial Hospital at Linkou Taoyuan Taiwan

**Keywords:** palliative chemotherapy, pancreatic cancer, S‐1, survival outcome

## Abstract

**Objective:**

Studies have rarely explored the efficacy of S‐1 in treating advanced pancreatic cancer outside Japan. This study compared the survival outcomes of patients with advanced pancreatic cancer treated with S‐1 with the survival outcomes of those without S‐1 treatment before and after S‐1 reimbursement was introduced in Taiwan in June of 2014.

**Method:**

We retrospectively analyzed 838 patients with locally advanced or metastatic pancreatic cancer who underwent palliative chemotherapy from 2010 to 2016 at 4 institutes in Taiwan. For survival analysis, patients were categorized into two groups according to whether they received S‐1 treatment as palliative chemotherapy after diagnosis: (a) S‐1‐treated (n = 335) and (b) non‐S‐1‐treated (n = 503) groups.

**Results:**

The median overall survival was longer in the S‐1‐treated group than in the non‐S‐1‐treated group (10.7 vs 6.0 mo, *P* < 0.001). Subgroup survival analyses showed that the S‐1‐treated group had more favorable outcomes than the non‐S‐1‐treated group in terms of stage III (19.6 vs 10.1 mo, *P* < 0.001) and stage IV (8.5 vs 5.3 mo, *P* < 0.001) disease. The disease control rates were 43.6% and 32.8% (*P* < 0.001) in patients treated with and without S‐1 in the first‐line setting, respectively. In multivariate analysis, exposure to S‐1 treatment was an independent prognosticator for survival.

**Conclusion:**

Our results support the clinical use of S‐1 as the treatment of choice for patients with locally advanced or metastatic pancreatic cancer, particularly in resource‐limited situations.

## 1 INTRODUCTION

Pancreatic cancer was the 12th most common cancer and the 7th leading cause of cancer death worldwide in 2015,^1^ and it has an extremely poor prognosis. Similarly, in Taiwan, pancreatic cancer was the 12th most common cancer and caused more than 2000 deaths in 2015.^2^ Epidemiological studies in Taiwan have shown a steady increase in the incidence rate of pancreatic cancer from 3.7 per 100 000 in 1999 to 5.0 per 100 000 in 2012.^3^, ^4^ Surgical resection is the only curative treatment modality for patients with localized pancreatic cancer.^5^ However, the majority (>80%) of patients present with unresectable locally advanced or metastatic pancreatic cancer at the time of diagnosis.^4^, ^6^, ^7^, ^8^ Chemotherapy is the treatment of choice for unresectable pancreatic cancer; however, the corresponding survival outcomes are unsatisfactory, with the 5‐year survival rate being only 8.5% in the Unites States^1^ and 6.7% in Taiwan.^4^

S‐1 is an oral 5‐FU derivative with tumor‐selective cytotoxicity. S‐1 has been widely used in Japan for treating various types of solid cancer since 1996 and received approval in Japan in 1999.^9^ A randomized phase III Gemcitabine and S‐1 Trial (GEST) study was conducted in both Japan and Taiwan to compare the clinical efficacy of S‐1 monotherapy, gemcitabine monotherapy, and S‐1 and gemcitabine combined therapy as first‐line chemotherapy for advanced pancreatic cancer.^10^ The study results revealed that the survival difference between the three treatment groups was nonsignificant. However, S‐1 monotherapy was not inferior to gemcitabine monotherapy in terms of overall survival (OS; median 9.7 vs 8.8 mo); moreover, the response rate in the S‐1‐treated arm was significantly higher than that in the gemcitabine‐treated arm (21% vs 13%), but grade 3 or 4 hematologic toxicity in the S‐1‐treated arm was less than that in the gemcitabine‐treated arm.^10^

In Taiwan, gemcitabine monotherapy or a gemcitabine–platinum combination regimen^11^ has become treatment of choice for advanced pancreatic cancer since the National Health Insurance (NHI) program started providing reimbursement for it in 2003. Although the mentioned phase III studies involving erlotinib,^12^ nab‐ paclitaxel,^13^ and FOLFIRINOX^14^ reported positive results, Taiwan's NHI program did not provide reimbursement for use of these novel agents in advanced pancreatic cancer treatment because of their marginal efficacy,^12^ problematic toxicity profiles,^14^ and concerns related to cost–benefit effects.^13^, ^15^ Nevertheless, on the basis of the GEST study results revealing that S‐1 was not inferior to gemcitabine monotherapy in terms of OS,^10^ the NHI program began providing reimbursement for S‐1 used in treating advanced pancreatic cancer in June of 2014. Compared with gemcitabine, S‐1 is more convenient to use in treatment. Therefore, patients with pancreatic cancer are increasingly gaining access to S‐1 treatment in Taiwan. Studies have rarely explored the efficacy of S‐1 in treating advanced pancreatic cancer outside Japan. Accordingly, the aim of the present study was to compare the survival outcomes of patients with advanced pancreatic cancer treated with S‐1 with the survival outcomes of those treated without S‐1 before and after the NHI program began providing reimbursement for S‐1 in Taiwan in June of 2014.

## 2 PATIENTS AND METHODS

### 2.1 Patient selection

We retrospectively reviewed the medical records of patients who received a new diagnosis of unresectable or metastatic pancreatic cancer from January, 2010 to December, 2016 at 4 branches of Chang Gung Memorial Hospital (CGMH) in Taiwan. All patients were either pathologically or radiographically diagnosed as having primary pancreatic cancer and had received palliative chemotherapy for more than 2 weeks after diagnosis. Patients who had tumor recurrence after radical surgery, experienced a concurrent active malignancy, had histology other than pathologically proven carcinoma subtypes, were enrolled in clinical trials for pancreatic cancer treatment, or were aged younger than 20 years were excluded. A total of 838 consecutive patients were included for final analysis. The chemotherapy regimens were determined by primary care physicians on the basis of the preference of patients and physicians. Patients were categorized into two groups according to whether they had received S‐1 treatment, regardless of S‐1 treatment lines, after the diagnosis of pancreatic cancer: (a) S‐1‐treated group (n = 335) and (b) non‐S‐1‐treated group (n = 503). Pretreatment clinical factors were analyzed in univariate and multivariate models for survival analysis. This study was approved by the institutional review boards of all CGMH branches and was conducted in compliance with the Declaration of Helsinki (1996).

### 2.2 Data collection

Using a prospectively formulated electronic data form from our previous research,^16^, ^17^ primary care physicians recorded the patients’ demographic and clinical data including age, sex, body mass index, Eastern Cooperative Oncology Group performance status (ECOG PS), smoking history, pre‐existing comorbidities as assessed using the modified Charlson comorbidity index (CCI),^18^ anatomic location of the primary cancer, clinical stage as determined using the 7th edition of the American Joint Committee on Cancer (AJCC), presence of drainage for obstructive jaundice, serum carcinoembryonic antigen (CEA) and carbohydrate antigen 19‐9 (CA19‐9) levels, organ of metastatic site, and chemotherapy regimens. Gemcitabine was administered at a dose of 1000 mg/m^2 ^on days 1, 8, and 15, and this cycle was repeated every 28 days.^19^ S‐1 was provided at a dose of 80‐120 mg on day 1 and day 14 in a 3‐week cycle or on day 1 and day 28 in a 6‐week cycle.^10^ The treatment regimen was determined by the primary care physician. Imaging studies were conducted during regular follow‐up every 3 months or were clinically indicated during the period of chemotherapy. Tumor response was evaluated through imaging studies according to the Response Evaluation Criteria in Solid Tumors (RECIST) 1.1. Patients who required early termination of chemotherapy, required a change of chemotherapy regimen, or died before imaging studies executed for response assessment were determined to have experienced disease progression. OS was calculated from the time of initiation of chemotherapy until the date of death from any cause. All included patients were followed until death or December 31, 2017. All dates of death were obtained from either the Institutional Cancer Registry or the National Registry of Death database in Taiwan.

### 2.3 Statistical analysis

Basic demographic data are presented as n (%) for categorical variables and median with range or 95% CI for continuous variables. The difference between the S‐1‐treated and non‐S‐1‐treated groups was determined using the Pearson chi‐squared (*χ* ^2^) test or Fisher's exact test if the number of variables in any cell was less than 5. The log‐rank test was used to perform univariate and multivariate analyses of OS for all clinical factors. All variables in the univariate analysis with *P *values of <0.15 were further analyzed using multivariate analysis. SPSS 17.0 software (SPSS Inc, Chicago, IL) was used for statistical analysis. All statistical assessments were 2 sided, and a *P* value of <0.05 was considered statistically significant.

## 3 RESULTS

### 3.1 Basic patient characteristics

The patient characteristics are summarized in Table 1. In the overall cohort, the median age was 63 years (range, 23‐89 years), and 59.3% of the patients were men. Most patients had stage IV pancreatic cancer (n = 655, 78.2%), and the most common metastatic sites were the liver (52.3%), peritoneum (28.5%), and distant lymph nodes (17.9%). Patients in the S‐1‐treated group typically had significantly better physical conditions (as indicated by ECOG PS scores of 0‐1; 80.9% vs 64.8%, *P* < 0.001), a higher prevalence of primary tumors of the pancreatic body (25.1% vs 12.7%, *P* < 0.001), a lower percentage of obstructive jaundice requiring drainage (26.6% vs 36.3%, *P* = 0.004), and more lines of palliative chemotherapy for pancreatic cancer (median 2 vs 1 line, *P* < 0.001) compared with those in the non‐S‐1‐treated group. No statistically significant differences in age, sex, body mass index (BMI), comorbidity, smoking history, tumor stage, abnormal CEA or CA19‐9 levels, organ of metastasis, previous surgery for primary cancer, or treated with radiotherapy were observed between the S‐1‐treated and non‐S‐1‐treated groups.

**Table 1:** Patients’ demographic data

Group	Overall n = 838	Without S1group n = 503	With S1group n = 335	*P* value
Median age, year (range)	63 (23‐89)	63 (25‐89)	62 (23‐88)	0.68
Sex, male	497 (59.3)	311 (61.8)	186 (55.5)	0.07
BMI, median (range)	22.5 (13.0‐36.2)	22.6 (13.0‐36.2)	22.3 (15.6‐36.0)	0.75
ECOG PS				
0‐1	597 (71.2)	326 (64.8)	271 (80.9)	<0.001
2	206 (24.6)	152 (30.2)	54 (16.1)	
3	35 (4.2)	25 (5.0)	10 (3.0)	
Smoking	306 (36.5)	171 (34.0)	135 (40.3)	0.07
CCI				0.10
0	227 (27.1)	130 (25.8)	97 (29.0)	
1	292 (34.8)	180 (35.7)	112 (33.5)	
2	193 (23.0)	109 (21.7)	84 (25.1)	
≥3	126 (22.3)	84 (16.7)	42 (12.6)	
7th AJCC Stage				0.27
III	183 (21.8)	97 (19.2)	86 (25.7)	
IV	655 (78.2)	406 (80.7)	249 (74.3)	
Primary tumor site				<0.001
Head	343 (40.9)	220 (43.7)	123 (36.8)	
Body	148 (17.7)	64 (12.7)	84 (25.1)	
Tail	171 (20.4)	100 (19.8)	71 (21.3)	
Overlapping	176 (21.0)	119 (23.6)	57 (17.1)	
Organ of metastases				
Liver	438 (52.3)	270 (53.7)	168 (50.1)	0.29
Peritoneum	239 (28.5)	156 (31.0)	83 (24.9)	0.06
Lung	98 (11.7)	58 (11.5)	40 (12.0)	0.83
CEA, median (range)	5.4 (0.5‐5892)	5.8 (0.5‐3310)	4.5 (0.3‐5892)	0.27
CA19‐9, median (range)	779 (0.6‐50000)	966.5 (0.6‐50000)	582 (0.5‐50000)	0.11
Biliary drainage	272 (32.5)	183 (36.3)	89 (26.6)	0.004
Previous surgery for primary cancer	31 (3.7)	22 (4.4)	9 (2.9)	0.26
Treated with radiotherapy	130 (15.5)	70 (13.9)	60 (17.9)	0.12
Use of chemotherapy agent				
Gemcitabine	792 (94.5)	491 (97.6)	301 (89.9)	<0.001
Platinum	433 (51.7)	268 (53.3)	165 (49.3)	0.26
S‐1	335 (40.0)	0	335 (100)	–
Line of chemotherapy				
Median (range)	1 (1‐7)	1 (1‐4)	2 (1‐7)	<0.001
1	486 (58.0)	368 (73.2)	118 (35.2)	
2	236 (28.2)	98 (19.5)	138 (41.2)	
3	98 (11.7)	34 (6.8)	64 (19.1)	
>3	18 (2.1)	3 (0.6)	15 (4.5)	

Note

BMI, body mass index; ECOG PS, Eastern Cooperative Oncology Group performance status; CCI, Charlson comorbidity index; AJCC, American Joint Committee on Cancer; CEA, carcinoembryonic antigen, CA19‐9, Carbohydrate cell surface antigen19‐9.

### 3.2 Survival outcomes

**Figure 1:**
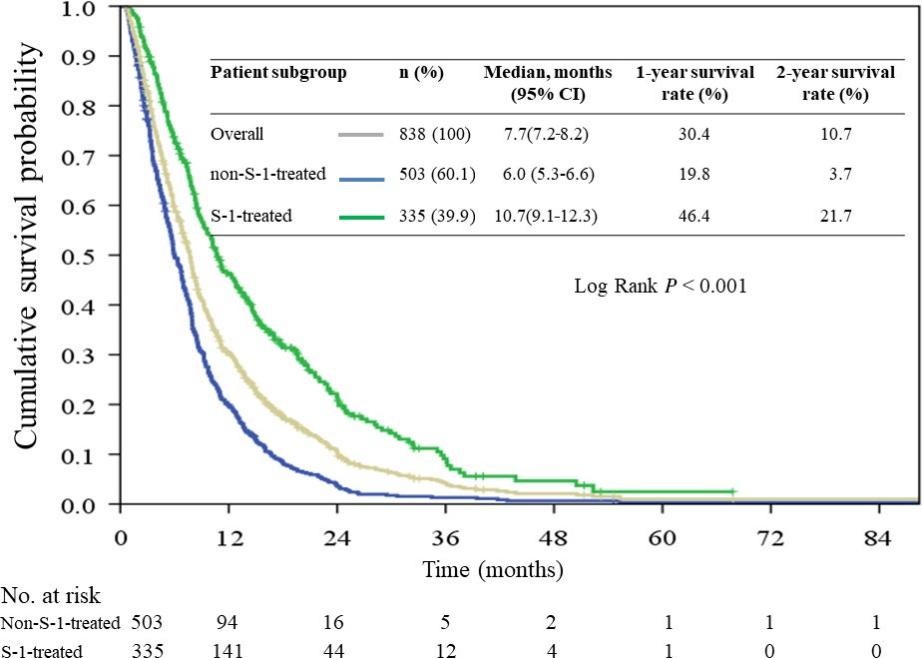
Overall survival curve

The median follow‐up time for the survivors was 13.8 (range, 0.6‐67) months, and 754 patients (90.0%) had died by the end of the study. The median survival times in the overall cohort, non‐S‐1‐treated group, and S‐1‐treated group were 7.7 (95% CI: 7.2‐8.2), 6.0 (95% CI: 5.3‐6.6), and 10.7 (95% CI: 9.1‐12.3) months, respectively (Figure 1). Patients in the S‐1‐treated group had a significantly longer survival time than those in the non‐S‐1‐treated group did (hazard ratio: 0.48, 95% CI: 0.41‐0.56, *P* < 0.001).

**Figure 2:**
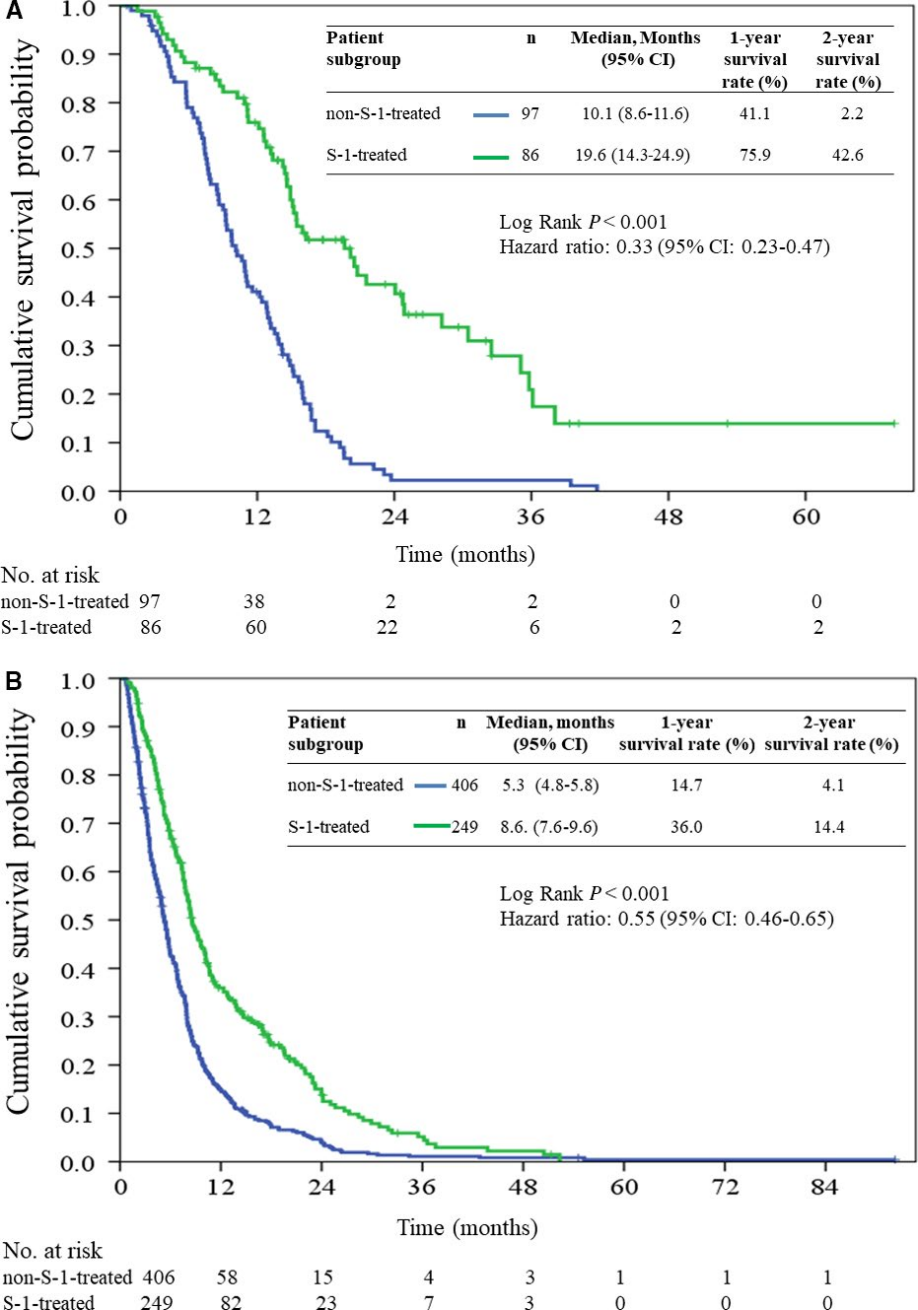
Kaplan‐Meier estimates of overall survival in stage III disease (A) and stage IV disease (B)

Subgroup survival analyses conducted according to AJCC stage showed that patients in the S‐1‐treated group had a more favorable outcome than those in the non‐S‐1‐treated group did for both stage III (Figure 2A) and stage IV (Figure 2B) pancreatic cancer.

### 3.3 Univariate and multivariate analyses for survival outcome

The clinical variables were subjected to univariate and multivariate analyses for predicting OS, and Table 2 presents the analysis results. The univariate analysis revealed that a younger age (hazard ratio 1.009 per 1 year increase in age, *P* = 0.007), female sex (vs male sex, *P* < 0.001), nonsmoking history (vs smoking history, *P* = 0.003), an ECOG PS score of 0‐1 (vs PS score of 2, *P* < 0.001; vs PS score of 3, *P* < 0.001), a CCI score of 0 (vs CCI score of 1‐2, *P* < 0.001; vs CCI score of ≥ 3, *P* < 0.001), AJCC stage III (vs stage IV, *P* < 0.001), CEA level of < 5.3 ng/mL (vs ≥ 5.3 ng/mL, *P* < 0.001), CA19‐9 level of < 780 ng/mL (vs ≥ 780 ng/mL, *P* < 0.001), gemcitabine treatment exposure (vs non‐gemcitabine treatment exposure, *P* < 0.001), Platinum treatment exposure (vs non‐platinum treatment exposure, *P* < 0.001), and S‐1 treatment exposure (vs non‐S‐1 treatment exposure, *P* < 0.001) were significantly associated with more favorable OS. Furthermore, based on the multivariate analysis, sex, smoking history, ECOG PS, CCI, AJCC stage, CA19‐9 level, gemcitabine treatment, platinum treatment, and S‐1 treatment were identified as adequate independent prognostic factors for OS.

**Table 2:** Univariate and multivariate analyses for overall survival

Variable	Category	n (%)	Overall survival in months, median (95%CI)	Univariate analysis		Multivariate analysis	
				HR (95% CI)	p	HR (95%CI)	*P*
Age	Years, 1/increase			1.009 (1.003‐1.016)	0.007	1.00 (0.99‐1.002)	0.14
Sex	Male	497 (59.3)	7.0 (6.4‐7.6)	1		1	
	Female	341 (40.7)	8.5 (7.8‐9.2)	0.76 (0.66‐0.89)	<0.001	0.81 (0.70‐0.94)	0.005
BMI	<23	474 (56.6)	7.2 (6.5‐7.9)	1			
	≥23	364 (43.4)	8.0 (7.2‐8.9)	0.93 (0.80‐1.07)	0.31		
Smoking	No	532 (63.5)	8.1 (7.5‐8.6)	1		1	
	Yes	306 (36.5)	6.7 (5.8‐7.7)	1.25 (1.08‐1.44)	0.003	1.29 (1.10‐1.50)	0.001
ECOG PS	0‐1	597 (71.2)	9.2 (8.5‐9.9)	1		1	
	2	206 (24.6)	4.0 (3.5‐4.5)	2.58 (2.18‐3.05)	<0.001	1.79 (1.48‐2.16)	0.005
	3	35 (4.2)	2.6 (1.9‐3.3)	4.46 (3.09‐6.45)	<0.001	3.11 (2.09‐4.63)	<0.001
CCI	0	227 (27.1)	9.5 (8.3‐10.7)	1		1	
	1‐2	485 (57.9)	7.5 (7.0‐8.0)	1.44 (1.21‐1.71)	<0.001	1.36 (1.10‐1.70)	0.002
	≥3	126 (22.3)	5.4 (4.5‐6.3)	1.92 (1.53‐2.42)	<0.001	1.84 (1.44‐2.35)	<0.001
AJCC stage	III	183 (21.8)	13.4 (11.9‐14.8)	1		1	
	IV	655 (78.2)	6.6 (6.0‐7.2)	1.99 (1.66‐2.39)	<0.001	2.15 (1.78‐2.59)	<0.001
Biliary drainage	No	566 (67.5)	7.7 (7.1‐8.3)	1			
	Yes	272 (32.5)	7.4 (6.4‐8.4)	1.11 (0.95‐1.29)	0.19		
CEA level, ng/mL	≤5.3	408 (48.7)	8.5 (7.6‐9.3)	1		1	
	>5.3	438 (51.3)	6.7 (5.9‐7.6)	1.31 (1.14‐1.52)	<0.001	1.13 (0.97‐1.31)	0.11
CA19‐9 level, u/mL	≤780	404 (48.2)	8.7 (7.8‐9.5)	1		1	
	>780	434 (51.8)	6.7 (5.9‐7.5)	1.38 (1.19‐1.59)	<0.001	1.18 (0.99‐1.42)	0.069
S‐1 treatment	No	503 (60.0)	6.0 (5.3‐6.6)	1		1	
	Yes	335 (40.0)	10.7 (9.1‐12.3)	0.48 (0.41‐0.56)	<0.001	0.48 (0.41‐0.56)	<0.001
Gemcitabine treatment	No	46 (5.5)	3.8 (3.1‐4.4)	1		1	
	Yes	792 (94.5)	7.7 (7.2‐8.2)	0.50 (0.37‐0.68)	<0.001	0.53 (0.39‐0.74)	<0.001
Platinum treatment	No	405 (48.3)	5.5 (4.9‐6.0)	1		1	
	Yes	433 (51.7)	9.2 (7.2‐8.2)	0.62 (0.53‐0.71)	<0.001	0.61 (0.52‐0.72)	<0.001
Previous surgery for primary cancer	No	807 (96.3)	7.6 (7.1‐8.1)	1			
	Yes	31 (3.7)	9.7 (4.7‐14.7)	0.79 (0.60‐1.15)	0.11		
Radiotherapy	No	708 (84.5)	7.8 (7.2‐8.4)	1			
	Yes	130 (15.5)	7.6 (6.9‐8.3)	0.91 (0.78‐1.05)	0.20		

Note

BMI, body mass index; ECOG PS, Eastern Cooperative Oncology Group performance status; CCI, Charlson comorbidity index; AJCC, American Joint Committee on Cancer; CEA, carcinoembryonic antigen; CA19‐9, Carbohydrate cell surface antigen19‐9; HR, hazard ratio.

### 3.4 Treatment regimens for patients in S‐1‐treated and non‐S‐1‐treated groups

**Figure 3:**
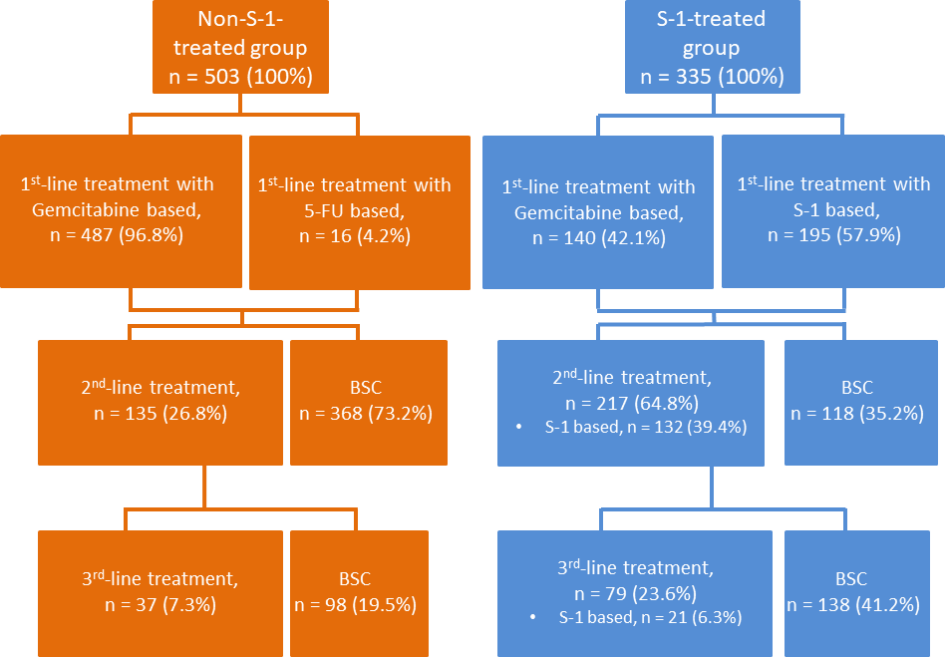
Treatment regimens for S‐1‐treated and non‐S‐1‐treated groups (G: gemcitabine, 5‐FU: 5‐fluorouracil, BSC: best supportive care)

Figure 3 illustrates the details of chemotherapy regimens chosen in different treatment lines for patients in the S‐1‐treated and non‐S‐1‐treated groups. Of the 503 patients in the non‐S‐1‐treated group, 468 (96.8%) received a gemcitabine‐based regimen as the first‐line palliative chemotherapy for pancreatic cancer, whereas the remaining patients (n = 16, 4.2%) received 5‐FU‐based regimens for treating pancreatic cancer. After the first‐line chemotherapy, 368 patients (73.2%) received best supportive care without further antitumor treatment. Only 135 (26.8%) and 37 (7.3%) patients received second‐ and third‐line treatment for pancreatic cancer, respectively. Regarding patients in the S‐1‐treated group, 195 (57.9%), 132 (39.4%), and 21 (6.3%) patients received S‐1‐containing regimens as the first‐, second‐, and third‐line treatment for pancreatic cancer, respectively. Overall, 64.8% and 23.6% of the patients in the S‐1‐treated group received second‐ and third‐line treatment for pancreatic cancer, respectively.

### 3.5 Best tumor responses according to treatment lines

**Figure 4:**
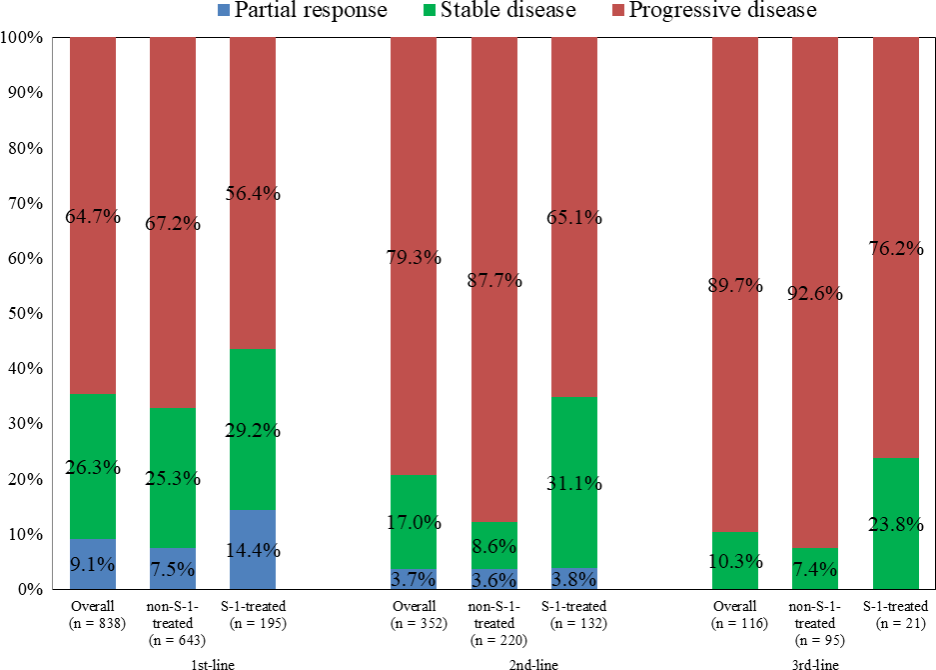
Best tumor responses of S‐1‐treated and non‐S‐1‐treated groups according to lines of palliative chemotherapy (PD: progressive disease, SD: stable disease, PR: partial response)

Figure 4 presents the best tumor responses among patients receiving chemotherapy; the responses were determined according to treatment lines. Overall, the rates of partial response and stable disease were, respectively, 9.1% and 26.3% in patients receiving first‐line chemotherapy, 3.7% and 25.3% in those receiving second‐line chemotherapy, and 0% and 10.3% in those receiving third‐line chemotherapy. In the first‐line setting, treatments containing S‐1 achieved significantly higher rates of partial response (14.4% vs 7.4%) and stable disease (29.2% vs 25.3%) than did those without S‐1. In the second‐line setting, the partial response rates were similar between the S‐1‐treated and non‐S‐1‐treated groups (3.8% vs 3.6%), but the stable disease rate in the S‐1‐treated group was still higher than that in the non‐S‐1‐treated group (31.1% vs 8.6%). None of the patients experienced partial response to chemotherapy in the third‐line setting; the stable disease rate in the S‐1‐treated group was more than 3 times higher than that in the non‐S‐1‐treated group (23.8% vs 7.4%).

## 4 DISCUSSION

The current study is the largest investigation conducted outside Japan to evaluate the effect of S‐1 on survival outcomes in patients with advanced pancreatic cancer. We demonstrated that stage III disease, female sex, nonsmoking history, favorable ECOG PS, no comorbidity, and receiving S‐1 treatment were suitable independent prognostic factors for survival outcome in advanced pancreatic cancer. In the first‐line chemotherapy setting, exposure to S‐1 treatment was associated with greater clinical efficacy in terms of OS (median, 10.7 vs 6.0 mo, *P* <0.001) and disease control rate (43.6% vs 32.8%, *P* < 0.001) compared with lack of exposure to S‐1 treatment. In addition, patients who received S‐1 treatment had a higher probability of receiving more lines of palliative chemotherapy for pancreatic cancer compared with those without S‐1 treatment (median, 2 vs 1 line, *P* < 0.001). Accordingly, our findings suggested that S‐1 exhibited a positive effect in prolonging survival outcomes in advanced pancreatic cancer in Taiwan.

Nearly all patients in the non‐S‐1‐treated group received gemcitabine monotherapy, gemcitabine plus cisplatin, or 5‐FU as the first‐line chemotherapy regimen for pancreatic cancer. Although gemcitabine monotherapy demonstratedgreater efficacy than 5‐FU did in improving survival in patients with advanced pancreatic cancer,^19^ the survival outcomes were still extremely poor, with the tumor response rate being 6%–11% and median OS time being 5.6‐8.8 months.^10^, ^12^, ^13^, ^19^, ^20^ Gemcitabine in combination with either capecitabine^21^, ^22^ or cisplatin^23^ has been reported to be more effective than gemcitabine monotherapy in prolonging progression‐free survival; however, no randomized trial has demonstrated significant survival differences.^21^, ^22^ The survival outcome (6.0 mo) and best tumor response rate (7.4%) observed in the patients in the non‐S‐1‐treated group in the present study were consistent with those observed in patients receiving gemcitabine monotherapy or gemcitabine plus cisplatin in previous studies.^12^, ^13^, ^19^, ^20^, ^23^ Our results indicated the clinical limitations of gemcitabine monotherapy, gemcitabine plus cisplatin, and 5‐FU treatments for advanced pancreatic cancer in daily practice.

Several studies conducted in Japan have revealed that S‐1 monotherapy was effective in treating advanced pancreatic cancer^24^, ^25^ and that combination therapy involving S‐1 and gemcitabine was associated with high antitumor activity.^26^, ^27^ A retrospective observational study revealed a significant improvement in survival outcomes in patients with advanced pancreatic cancer before and after the introduction of S‐1 in Japan.^31^ Similarly, our study showed significant survival benefits in patients treated with S‐1 compared with those without S‐1 treatment. The median survival time was 10.7 months in patients in the S‐1‐treated group in our study, which was comparable to the median survival times in patients who received S‐1 treatment (9.7 mo) and gemcitabine plus S‐1 treatment (10.1 mo) in the GEST study. However, the objective response rate (14.4%) among our patients who received S‐1 as the first‐line treatment was lower than that in patients who received S‐1 monotherapy in the GEST study (23.8%)^10^; this difference may have been related to the different general conditions of patients in the real world. Our results demonstrated that the partial response and stable disease rates in patients who received S‐1 as a first‐line treatment were higher than those in patients who did not receive S‐1 as a first‐line treatment. This finding may partially explain the survival difference observed between our patient groups.

In our patient cohort, 135 of the 503 (26.8%) patients in the non‐S‐1‐treated group and 217 of the 335 (64.8%) patients in the S‐1‐treated group received second‐line chemotherapy for advanced pancreatic cancer. Of the 217 patients in the S‐1‐treated group, 132 (60.8%) received S‐1‐based agents as second‐line treatment regimens. A phase III study was recently conducted in Japan and Korea to compare S‐1 monotherapy with S‐1 plus leucovorin as second‐line regimens for treating gemcitabine‐refractory pancreatic cancer.^32^ The regimens engendered similar survival outcomes (7.9 mo in S‐1 group, 7.6 mo in S‐1 plus leucovorin group, *P* = 0.76). The S‐1 group exhibited a notable tumor response rate (15.1%) and stable disease rate (44.1%).^32^ Similarly, a retrospective analysis conducted in Japan demonstrated that administering S‐1 as second‐line therapy for treating gemcitabine‐refractory pancreatic cancer resulted in a partial response rate of 17.2% and OS of 7.7 months.^33^ The author of this retrospective analysis reported that the percentage of patients receiving second‐line treatment was significantly elevated from 12.8% before the introduction of S‐1 to 45.9% after the introduction of S‐1. Accordingly, the survival time from gemcitabine failure to death was prolonged from 3.1 months before S‐1 was introduced to 6.7 months after S‐1 was introduced (*P* < 0.001) in their institute.^33^ Consistent with the findings of previous studies,^32^, ^33^ our results indicated that S‐1 was effective as a sequential therapy in treating gemcitabine‐refractory pancreatic cancer; patients’ survival was prolonged after the introduction of S‐1 in Taiwan.

We calculated the OS from the date of initiating palliative chemotherapy to the date of death to be consistent previous reports.^31^, ^33^ Our intent is to evaluate the impact of chemotherapy on survival in patients with advanced pancreatic cancer. The lapse between the diagnosis and initiation of chemotherapy was 15.0 (95% CI: 12.6‐17.3) days in the non‐S‐1‐treated group and 13.5 (95% CI: 11.6‐15.7, *P* = 0.08) days in the S‐1‐treated group. Either calculating the OS from the date of diagnosis or the date of chemotherapy initiation did not change the result in survival differences between groups.

According to our review of the literature, this study is the largest population‐based series including patients from 4 institutes across Taiwan over a 7‐year period to demonstrate the effect of S‐1 introduction on survival outcomes in patients with advanced pancreatic cancer. Nevertheless, several limitations should be addressed. First, selection bias might have occurred in this retrospective study. The patients’ characteristics were not equally distributed in each group. For example, the S‐1‐treated group included more patients with ECOG PS scores of 0‐1 (80.9% vs 64.8%) and smoking history (40.3% vs 34.0%) but included fewer patients with comorbidities (71.0% vs 74.2%), peritoneal metastasis (24.9% vs 31.0%), and biliary drainage (26.6% vs 36.3%) than the non‐S‐1‐treated group did. Despite these discrepancies between the 2 groups, S‐1 treatment had a significant effect on survival outcomes in the multivariate analysis, and this effect persisted after adjustment for the other confounding factors. Second, no central review was established for the imaging studies; this may have resulted in overestimations of the tumor responses in our study. Third, some of our patients received S‐1 treatment before the NHI program provided reimbursement for such treatment, and the relatively high financial status of such patients may have affected the survival outcomes. In addition, the S‐1 treatment in our study was given at 80‐120mg on day 1 and day 14 in a 3‐week cycle or on day 1 and day 28 in a 6‐week cycle.^10^ The designation to either a 3‐week or 6‐week cycles was made by individual primary care physicians. Our study did not include the variable of S‐1 treatment schedule in survival analysis for which might influence the statistical outcome. Fourth, the use and efficacy of oral medications, including S‐1, might be limited in patients with bowel obstruction or those receiving enteral tube feeding. Because of the lack of data regarding patients with bowel obstruction or those receiving enteral tube feeding, we could not evaluate the prognostic effect of S‐1 in such patients. Finally, the health care system in Taiwan differs from those in other countries in that the Taiwanese system does not provide reimbursement for use of erlotinib, nab‐paclitaxel, oxaliplatin, or irinotecan in treating pancreatic cancer. Hence, the results of this study may not be suitable for general clinical application in treating patients with pancreatic cancer worldwide. However, our results revealed that the use of S‐1 in anticancer treatment could benefit OS in patients with advanced pancreatic cancer and may be considered as an alternative regimen in resource‐limited situations. Further investigation should be conducted on the incorporation of S‐1 into the treatment course for patients with advanced pancreatic cancer treated with nab‐paclitaxel or FOLFIRINOX as frontline regimens.

## 5 CONCLUSION

This study is the largest population‐based investigation conducted outside Japan to demonstrate the effect of S‐1 on survival outcomes in patients with advanced pancreatic cancer. Patients with advanced pancreatic cancer who received S‐1 treatment exhibited significantly higher OS and disease control rates than patients without S‐1 treatment did. Our results suggested that S‐1 had a positive effect in prolonging the survival outcomes of pancreatic cancer in Taiwan. The results supported the use of S‐1 as the treatment of choice in clinical practice for treating patients with locally advanced and metastatic pancreatic cancer, particularly in resource‐limited situations.

## CONFLICTS OF INTEREST

No competing financial interests exist.
